# Prejudice and Ethnic Bullying Among Children: The Role of Moral Disengagement and Student-Teacher Relationship

**DOI:** 10.3389/fpsyg.2021.713081

**Published:** 2021-09-03

**Authors:** Nicolò Maria Iannello, Marina Camodeca, Carmen Gelati, Noemi Papotti

**Affiliations:** ^1^Department of Languages and Literatures, Communication, Education, and Society, University of Udine, Udine, Italy; ^2^Department of Psychology, University of Milano – Bicocca, Milan, Italy; ^3^Department of Psychology, Catholic University of the Sacred Heart, Milan, Italy

**Keywords:** ethnic prejudice, ethnic bullying, moral disengagement, closeness with the teacher, primary school

## Abstract

The identification of factors associated with ethnic bullying within multiethnic schools is a timely social issue. Up to now, ethnic prejudice has been found to facilitate aggression triggered by schoolmates’ cultural background. Yet, there is still a dearth of research about the mechanisms underlying this relation among children. In order to fill this gap, by adopting a social-cognitive developmental perspective on prejudice and morality, this paper investigated the mediating role of moral disengagement in the association between ethnic prejudice and ethnic bullying, as well as the moderating role of closeness with the teacher. A mediation model and a moderated mediation model were applied to data collected from 552 primary school children aged 8–10years. Ethnic prejudice, ethnic bullying, and moral disengagement were assessed through self-reported questionnaires, whereas a questionnaire was administered to teachers to assess the level of closeness with their pupils. Results indicated that ethnic prejudice was directly and positively related to ethnic bullying and that moral disengagement partially mediated this association. This indirect link was particularly strong for children with low levels of closeness with their teachers, whereas it resulted not significant for pupils with high levels of closeness, suggesting that closeness with the teacher might restrain morally disengaged children from enacting ethnic bullying. Implications for research and practice aimed at reducing prejudice and moral disengagement, as well as at promoting positive relationships among children and between pupils and teachers, are discussed.

## Introduction

The term ethnic bullying identifies an aggressive action perpetrated toward individuals on ground of their ethnic origins (Elamé, 2013). Similar to traditional bullying, this type of bias-based harassment is carried out, intentionally and repetitively, against children who are not able to defend themselves and is enacted through verbal attacks (e.g., name calling), physical means (e.g., hitting), and relational/social aggression (e.g., exclusion; McKenney et al., 2006; Scherr and Larson, 2010; Elamé, 2013).

Several studies highlighted the incidence of bullying related to ethnicity among school-age children in Europe (Strohmeier et al., 2011; Xu et al., 2020). For instance, in Italy, primary and middle school students belonging to minority groups are likely to be easy targets of aggression by majority group members (Caravita et al., 2016). Moreover, in Britain schools, Hindu, Indian Muslim, and Pakistani children have been found to be bullied by other Asian pupils because of differences in skin color, faith, spoken language, and/or traditions (e.g., food and clothing; Eslea and Mukhtar, 2000).

Negative consequences of ethnic bullying on individuals’ development are documented and include poor adjustment and internalizing and externalizing problems (McKenney et al., 2006). Yet, little is known about what might push or limit the attacks toward peers on ground of their ethnic background (Bayram Özdemir et al., 2015). A better understanding of ethnic bullying seems to be urgent in a country like Italy where non-Italian students, mainly belonging to a second generation of immigrants, are increasing in number and have a migratory background traceable in more than 200 countries (Italian Ministry of Education, 2020). Such a situation results in a mosaic of traditions and cultures requiring innovative practices aimed at promoting not only social inclusion of students with different ethnic background, but also positive relationships at school. From this perspective, it is useful identifying mechanisms and risk and protective factors that might encourage and/or restrain ethnic bullying.

## Ethnic Prejudice and Bullying

Ethnic prejudice refers to the tendency to overgeneralize and simplify (mostly in a negative sense) information on other cultural groups and to have irrational preconceptions about them (van Dijk, 1984). In particular, it relates to beliefs and thoughts about ethnically different groups or individuals (cognitive component), to the emotional reactions (e.g., discomfort) associated with these groups and individuals (affective component), and to the actions carried out toward these targets (behavioral component; Rosenberg and Hovland, 1960; Duckitt, 2003). Despite their young age, even children seem to hold negative views on outgroup members (Levy et al., 2004). Particularly, they seem to progressively shift from a condition in which they have a mere preference for their in-group to a phase in which they might adversely appraise outgroups (Nesdale, 2010).

Although it has been highlighted that hostile predispositions toward those who are culturally different are likely to drive bias-based bullying at school (Dessel, 2010), research so far mainly focused on adolescents (Bayram Özdemir et al., 2015; Caravita et al., 2020). Interestingly, some works have stressed that emotions, more than beliefs, are at stake when individuals relate to outgroup members and that the emotional component of prejudice might be conductive of ethnicity-based bullying among youth (Papotti and Caravita, 2020). In the present study, we borrowed from this body of works on adolescents and explored the role of children’s negative attitudes toward outgroups in fostering ethnic bullying. Particularly, given the relevant part of emotions in conditioning intergroup relations (Tropp and Pettigrew, 2005), the affective facet of children’s ethnic prejudice was investigated and hypothesized to have a direct association with ethnic bullying. In other words, it was anticipated that children who experience negative feelings toward culturally different groups are more likely to attack their members.

## Moral Disengagement, Ethnic Prejudice, and Bullying

In their pathway toward ethnicity-based bullying, children may also turn to specific moral cognitive distortions that would help them legitimate their reprehensible behaviors (Caravita et al., 2019). In particular, according to Bandura’s (1991) social-cognitive theory of moral thought and action, individuals tend to deactivate moral control over their conduct through moral disengagement, a means by which people avoid self-sanctions and negative emotions (e.g., guilt and shame) that would prevent them from engaging in harmful acts. Literature agrees that, despite individuals judge bullying as wrong, they continue carrying out different forms of harassment by condoning their behaviors through moral cognitive processes (Hymel and Bonanno, 2014; Killer et al., 2019; Lo Cricchio et al., 2020). In light of this, it is reasonable to suppose that moral disengagement over personal actions against culturally different peers may be directly linked to ethnic bullying (Caravita et al., 2019; Bayram Özdemir et al., 2020).

Although the relationship between ethnic prejudice and moral disengagement is an understudied topic, some findings showed positive correlations between prejudice and the mechanism of dehumanization (Costello and Hodson, 2012), that might indicate that children who have negative views about ethnically different peers may also be inclined to justify discrimination through moral cognitive distortions. In turn, the belief that members of the outgroup lack human attributes may be conductive of negative behaviors, as found among adults (Vaes et al., 2003; Demoulin et al., 2004). On the base of these findings, it was supposed that moral disengagement might mediate the relation between affective ethnic prejudice and ethnic bullying. Specifically, a way in which children might turn their negative feelings and attitudes into reprehensible behaviors is by condoning their actions through moral disengagement mechanisms, which may make their conduct appear legitimate and, thus, facilitate ethnic bullying. This line of reasoning may be justified by the integrative social-cognitive developmental perspective on prejudice (Rutland et al., 2010), which posited that children consider together group-based criteria (e.g., group identity, in-group favoritism, and stereotyping) and morality (e.g., believing that it is fair/unfair to exclude someone) when they are about to reject groups and individuals. Given that moral disengagement processes might be influenced by situational dimensions (e.g., targets’ immigrant status), in the current study, a measure of moral disengagement that assesses the proneness to justify transgressive behaviors toward peers with a different cultural background was used (Caravita et al., 2019).

## The Role of the Relationship with Teachers

Consistently with Bandura’s (1986) social-cognitive theory, moral behaviors are the product of the interactions between individual and environmental factors. In light of this, we may suppose that the effect of ethnic prejudice and moral disengagement on ethnic bullying at school might be damped by specific contextual factors, such as the student-teacher relationship. Indeed, teachers spend much time with their pupils, represent relevant adults for children, and are likely to affect their development and behaviors. In particular, they often play a protective role in face of different risks (Sabol and Pianta, 2012). Generally, the student-teacher bond is evaluated on the base of its quality, that is, the extent to which the dyad is characterized by close or conflictual interactions (Fraire et al., 2008; Sabol and Pianta, 2012).

Following a social learning framework (Bandura, 1971, 1986), children learn and behave through modeling and imitation of others, as well as through vicarious experiences, particularly when the role models are socializing agents (e.g., teachers and parents) or are taken as positive models. Therefore, children and adolescents “with positive social relationships with parents, peers, and teachers benefit from these experiences and, therefore, are more likely to display better social, emotional and behavioral outcomes” (Wachs et al., 2020, p. 2). In addition, teachers who hold a positive relationship with their pupils may be more prone to promote an efficacious communication with them, encourage them, reinforce positive behaviors, and provide helpful feedbacks, which may foster pupils’ self-efficacy and willingness to behave properly (Wachs et al., 2020).

Relationships with teachers might be viewed from an attachment perspective as well (Davis, 2003; Bouchard and Smith, 2017). Referring to Bowlby’s (1969) theory, a secure relationship with a caregiver, being parent or teacher, is predictive of psychosocial and emotional adaptation. Teachers may contribute to children’s working models of peer relationships, by fostering useful skills for self-regulation and child-to-child interactions, and by hindering aggressive interactions (Bouchard and Smith, 2017). A strong bond with significant others, such as teachers, may also increase students’ sense of being valued and trusted and facilitate social sharing of experiences and feelings, which may inhibit bullying behaviors (Cho and Lee, 2018; van Aalst et al., 2021). As a matter of fact, children securely attached with teachers are less likely to be involved in bullying (Cho and Lee, 2018). This protective role of reorganizing relational schemas is particularly relevant for those children with insecure previous experiences of attachment or problem behaviors, such as aggression, and compensates for negative relationships with peers (Sabol and Pianta, 2012; van Aalst et al., 2021).

Whatever mechanisms are involved, several studies pointed out that a close relationship with teachers is associated with fewer bullying episodes and its negative outcomes, whereas a conflictual relationship with teachers seems to increase bullying involvement (Richard et al., 2011; Wang et al., 2015; Longobardi et al., 2018; Camodeca and Coppola, 2019). Teachers seem to play an important role also in orienting their pupils’ morality and attitudes toward ethnic outgroups. In general, it has been shown that perceiving a positive school climate, that includes support from teachers, might weaken the impact of moral disengagement on students’ bullying perpetration (Teng et al., 2020). In particular, it could be surmised that teachers sharing close connections with their students might help them consider the consequences of immoral conducts, act properly by monitoring their cognitions and attitudes, and, consequently, prevent them from bullying others with different origins. In addition, recent studies pointed out that close relationships with the teachers, as secondary attachment figures, might provide children with a sense of relational security that would help them be more open to and have positive attitudes toward ethnic outgroups (Geerlings et al., 2017). In sum, on ground of these theoretical considerations and empirical evidences, it could be hypothesized that the effect of both ethnic prejudice and moral disengagement on ethnic bullying might vary as a function of the extent to which teachers have a warm relationship with their pupils. It could also be possible that a positive relationship between pupils and teachers might inhibit the path from ethnic prejudice to ethnic bullying by deactivating moral disengagement mechanisms.

## The Present Study

**Figure 1:**
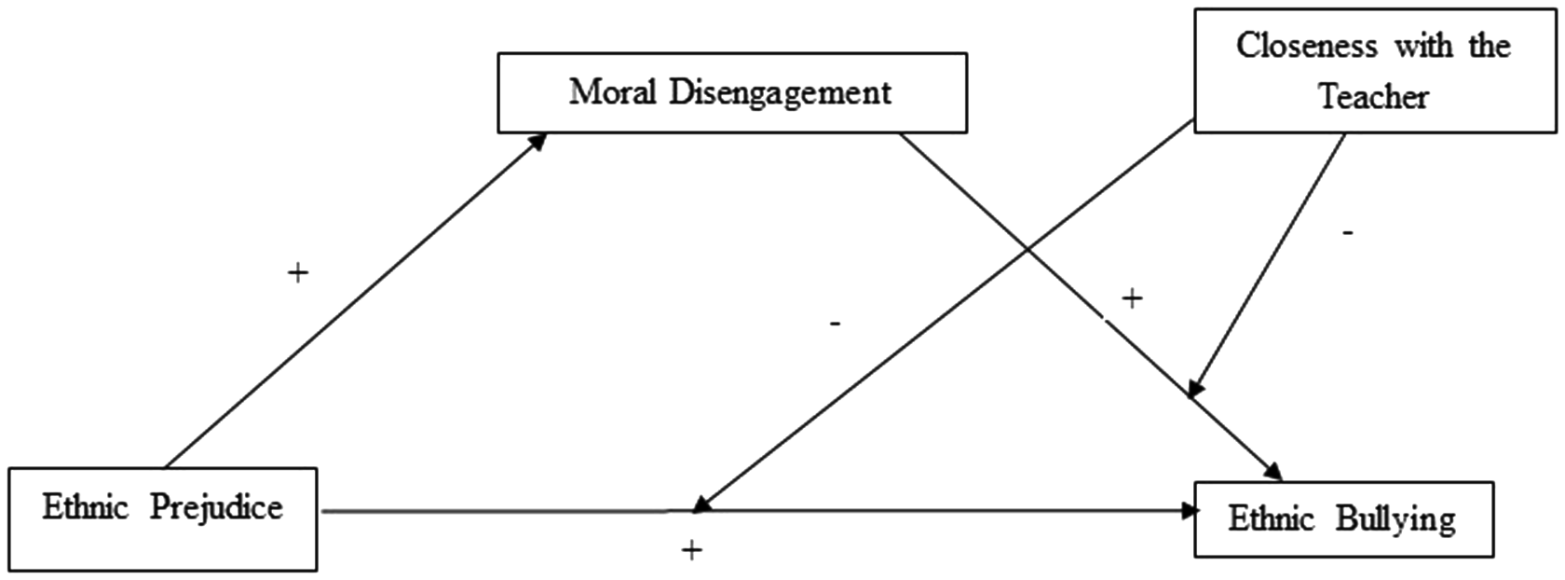
Theoretical model in which ethnic prejudice is expected to affect ethnic bullying through moral disengagement and in which closeness with the teacher is expected to moderate direct and indirect associations.

To the best of our knowledge, although both ethnic prejudice and moral disengagement have been found to be related to ethnic bullying (Bayram Özdemir et al., 2015; Caravita et al., 2019), and warm student-teacher interactions positively influence pupils’ cognitions and behaviors (Davis, 2003; Sabol and Pianta, 2012), all these variables have never been investigated within a comprehensive conceptual model. In order to fill this gap, the present work sought to provide a better understanding of how affective ethnic prejudice^1^, moral disengagement, and closeness with the teacher might jointly impact ethnicity-based bullying among primary Italian and immigrant school children. The links between these variables were tested within a moderated mediation model (Figure 1). Particularly, on the basis of the aforementioned discussion and literature, it was hypothesized that (H1) ethnic prejudice would be positively and directly associated with both ethnic bullying and moral disengagement; (H2) moral disengagement would be positively and directly associated with ethnic bullying; (H3) moral disengagement would mediate the relation between ethnic prejudice and ethnic bullying; and (H4) the quality of student-teacher relationship, considered as a protective factor, would moderate the direct and indirect associations between ethnic prejudice, moral disengagement, and ethnic bullying, which would be weaker for children with a positive relationship with their teachers.

Finally, gender, grade, and immigrant status were controlled for due to their role in affecting study variables, as indicated in the literature. Indeed, boys, compared to girls, have been found more prejudiced (Costello and Hodson, 2012), more prone to bully others on ground of their ethnicity (Bayram Özdemir and Özdemir, 2020), more morally disengaged (Thornberg and Jungert, 2014), and less close with their teachers (Murray and Murray, 2004). Younger children have been reported to be more prejudiced (Raabe and Beelmann, 2011) and to have warmer interactions with the teacher (Drugli, 2013) than older pupils. Students from minority groups, in comparison with the majority ones, have been found to show more problematic relationships with their teachers (Jerome et al., 2009) and higher levels of moral disengagement (Caravita et al., 2019), whereas findings about differences in ethnic bullying involvement between minority and majority groups are still scarce (Tolsma et al., 2013).

## Materials and Methods

### Participants

The sample included 552 children aged 8–10years (*M*=9.08years, *SD*=0.59; 289 boys and 263 girls). Pupils attended the third (44.9%) and the fourth grade (55.1%) of 13 public primary schools in two different areas in Northern Italy. The sample was ethnically heterogeneous with children mostly Italians^2^ (74.6%). Children from other countries were mainly born in Italy (82%), whereas their cultural background posed them as original from Eastern Europe (31%), North Africa (22%), Far East (14%), South America (11%), other African countries (10%), other European countries (9%), and other (3%). The sample included students from a wide range of sociocultural backgrounds (from low and working class to upper class), with a university degree obtained by 32% of fathers and 41% of mothers, whereas 20% of fathers and 14% of mothers did not obtained a high school degree.

### Procedure

The present study is part of a large project aimed at investigating ethnic bullying and including many measures. The main objectives and the methodology of the study were introduced to school principals and teachers, who agreed to participate. Parents were sent a letter to explain the study and were asked to give their informed consent, which was granted for 84.41% of the original sample contacted. The instruments were administered to students in the class group, during school time, in two different days. Children were first explained what we meant with “ethnic” or “origin,” saying that we referred to “those people (or their families) who talk a different language, or have the culture, the skin color, or the religion different from your own, or who come from different countries. For instance, we can think about ethnic groups such as Italians, Chinese, Albanians, Moroccans.” Teachers filled their questionnaires within 1week. Participants were assured about the confidentiality of all the information provided and that they could withdraw at any time. The Ethical Committee of the University of Udine approved this study, and all procedures were performed in accordance with the ethical principles for psychological research of the Italian Association of Psychology.

### Measures

#### Sociodemographic Variables

Participants’ sociodemographic variables were provided by their parents who were asked to indicate their children’s gender, age, grade, and place of birth. Parents were also asked to indicate their own countries of origin, education level, and job.

#### Ethnic Prejudice

The affective component of ethnic prejudice was assessed through two items aimed at measuring the extent (from 0 = very happy to 4 = very annoyed) to which respondents felt happy or annoyed to sit next to a classmate “from a different cultural background (e.g., with a different skin color or language) than your own” and “with a different religion from your own” (adapted; Buccoliero and Maggi, 2017).

#### Moral Disengagement

Children were proposed with the following hypothetical scenario, specifically designed to assess children’ proneness to legitimate negative behaviors toward a newcomer immigrant student (Caravita et al., 2019); the gender of the protagonist matched participants’ gender (“Hamir” in the male scenario and “Elissar” in the female scenario): *“Hamir/Elissar, a child from another country, is your new classmate; for some weeks you have both been back at school after the summer vacation. You started to call him/her “stupid” because he/she does not talk much; you also started to wait for him/her in the corridor before lessons and hit him/her to let him/her fall down. Sometimes you hide his/her school bag so that he/she could not find it and damage his/her books or copybooks. At the break, you do not talk with Hamir/Elissar and you do not want to play with him/her; you do not even want to invite him/her at your birthday party that you are organizing with your classmates. Hamir/Elissar in all these situations cannot defend him/herself.”* In order to assess self-justifications processes for harassing culturally different peers, children were asked to answer eight questions on a 5-point Likert-type scale, ranging from totally false (1) to totally true (5; e.g., “If you misbehave towards Elissar/Hamir it is because he/she misbehaved towards you first”; “Damaging books is not really harmful”). The wording of scenario and items was simplified to make it more suitable for young children. Items were averaged to rate moral disengagement as an overall disposition to condone one’s despicable behaviors; high scores indicate high moral disengagement.

#### Ethnic Bullying

An adaptation of the Florence Bullying and Victimization Scale (FBVS; Palladino et al., 2020) was used to evaluate ethnic bullying. A definition of bullying was presented prior to administering the scale *“Bullying happens when some children offend, ignore, kick, push, threaten, exclude other peers on purpose, or say bad things behind their back. It is also bullying when a child is teased repeatedly and in a nasty way. These episodes happen frequently, and it is difficult for the children who suffer from bullying to defend themselves. It is not bullying if two students of about the same strength quarrel or fight.”* Children were asked to think how often they have been involved in bullying behaviors in the last 2 or 3months. Despite the instrument assesses traditional bullying and victimization as well, for the purpose of the current study, four items concerning ethnic bullying perpetration were used, covering different forms of bullying (e.g., “I have hit/excluded/teased/spread rumors about/someone because of his/her origin, for instance for the color of the skin, the language, the religion”). Responses were on a 5-point Likert-type scale, from never (1) to several times a week (5).

#### The Quality of Student-Teacher Relationship

The Student-Teacher Relationship Scale (Pianta, 1994; Italian version by Fraire et al., 2008) was adopted to investigate how teachers perceived their relationship with each pupil. The instrument consists of three subscales: Closeness, Conflict, and Dependence. For the purpose of this study, only the Closeness subscale was used, which comprises eight items (e.g., “I share an affectionate, warm relationship with this child”). Teachers responded on a 5-point Likert-type scale, ranging from not applicable (1) to totally applicable (5).

The choice of considering only the Closeness scale mirrors our aim of testing a possible protective factor, discarding, therefore, the Conflict scale, which is usually regarded as a risk factor. In addition, the Dependence scale resulted unreliable in previous studies and, although it may be considered a protective factor at a young age, it includes aspects which can be risky at the end of primary school (Camodeca and Coppola, 2019).

### Data Analysis

Only data from students who were present on both administration days, or who had the opportunity to fill all questionnaires, were considered. Among these, missing data were very few in each item of study variables (range: 0.4–2.3%) and were handled using expectation maximization (EM) algorithm (Graham, 2009). As to categorical variables, only immigrant status had missing values (*n*=18; 3.2%) and participants with missing values did not significantly differ from those with complete data on any study variable, except for moral disengagement (*t*=2.34; *p*<0.05; *M*_Missing_=2.37 and *M*_Complete_=2.00). Following literature, we employed complete case analysis, removing these 18 participants, which is considered reasonable when missing data are less than 5% (Graham, 2009; Jakobsen et al., 2017).

Reliabilities were calculated as the greater lower bound (glb) index, indicating the lowest value of the real reliability (which ranges from glb to 1; Sijtsma, 2009). In order to examine the relations among study variables, Pearson correlations were employed. T-tests were conducted to compare boys and girls, younger and older children, and immigrant and non-immigrant children on the study variables.

The possibility of conducting a multilevel analysis was taken into account, given the nested nature of data. The intra-class correlation index (ICC) was calculated, which was below 0.05. Following the literature that identifies ICC indexes higher than 0.05 as suitable for performing a multilevel analysis, we decided to implement other types of analysis (Koo and Li, 2016).

Mediation and moderated mediation were tested by using PROCESS macro (Hayes, 2013), which calculates a series of regressions and includes all predictors in one block. Particularly, Model 4 was selected to test whether moral disengagement mediated the link between ethnic prejudice and ethnic bullying. Model 15 was employed to test whether closeness with the teacher moderated the association between ethnic prejudice and ethnic bullying via the mediator and whether the interactions terms between closeness with teachers and ethnic prejudice/moral disengagement affected ethnic bullying. Bootstrapping with 5,000 resamples to compute 95% confidence intervals was used to test the significance of the regression coefficients. If the confidence intervals did not contain zero, then statistics were significant. Gender, grade, and immigrant status were entered as covariates in all analyses.

## Results

### Preliminary Analyses

Table 1 summarizes means, standard deviations, reliabilities, and the correlation matrix. Positive and significant correlations emerged between ethnic prejudice, moral disengagement, and ethnic bullying perpetration. Closeness with the teacher was negatively correlated with ethnic prejudice and bullying.

**Table 1:** Descriptive statistics and bivariate correlations among study variables.

	1	2	3	4
1. Ethnic Prejudice	–			
2. Moral Disengagement	0.23^***^	–		
3. Ethnic Bullying	0.15^***^	0.15^***^	–	
4. Closeness with teacher	−0.14^**^	−0.04	−0.10^*^	
Means (SD)	0.83 (0.93)	2.00 (0.65)	1.08 (0.28)	4.00 (0.77)
Reliabilities	0.73	0.65	0.83	0.93

Reliabilities indicate the greater lower bound (glb) index

* *p*<0.05;

** *p*<0.01;

*** *p*<0.001.

*T*-tests indicated that girls had warmer relationships with their teachers (*t*=−3.55; *p*<0.001) and less prejudice (*t*=3.28; *p*<0.01) than boys. Also, third graders had closer relationships with their teachers than fourth graders (*t*=3.72; *p*<0.001); moreover, students from a migratory background, compared to their Italian peers, were more prone to bully others on ground of their ethnicity (*t*=−2.46; *p*<0.05) and were less close to their teachers (*t*=2.01; *p*<0.05).

### Mediation Effect Analysis

**Figure 2:**
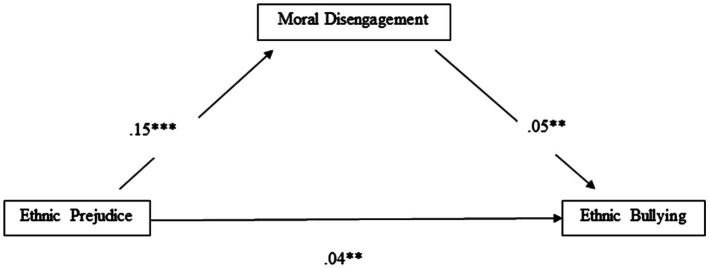
Unstandardized regression coefficients of the mediation model. The covariates (gender, grade, and immigrant status) were not included in the figure. The indirect effect of ethnic prejudice on ethnic bullying via moral disengagement was also significant (*B*=0.01; *SE*=0.01, 95% *CI*=0.0003, 0.0197). **p<0.01; ***p<0.001.

Model 4 in PROCESS macro (Hayes, 2013) was used to test the mediating effect of moral disengagement on the link between ethnic prejudice and ethnic bullying perpetration, while controlling for gender, grade, and immigrant status. The results are shown in Figure 2. Ethnic prejudice was significantly and positively related to moral disengagement (*R*^2^=0.05; *p*<0.001) and both ethnic prejudice and moral disengagement had a significant positive association with ethnic bullying (*R*^2^=0.05; *p*<0.001). In addition, the indirect effect of ethnic prejudice on ethnic bullying through moral disengagement was also significant (*B*=0.01; *SE*=0.01; 95% *CI*=0.0003, 0.0197). Gender and grade were not significantly related to any of the model variables, whereas immigrant status was positively associated with ethnic bullying (*B*=0.04; *p*<0.01), indicating that children with a migratory background were more involved in ethnic bullying than their Italian peers.

### Moderated Mediation Effect Analysis

**Figure 3:**
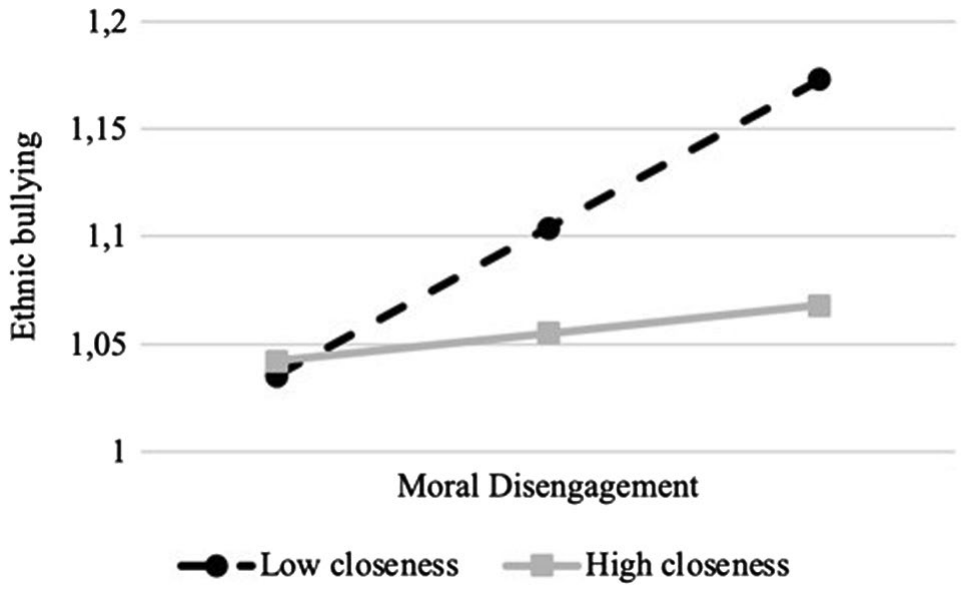
Interaction between moral disengagement and closeness with the teacher on ethnic bullying.

Closeness with the teacher was expected to moderate the associations between ethnic prejudice and ethnic bullying and between moral disengagement and ethnic bullying, as well as the indirect link. To test this hypothesis, while controlling for gender, grade, and immigrant status, closeness with the teacher was included and Hayes’ s PROCESS macro (model 15) was used. As Table 2 illustrates, with regard to covariates, as in the previous analysis, only immigrant status was significantly and positively associated with ethnic bullying, indicating a higher involvement for immigrant than Italian children. Closeness with the teacher was not associated with ethnic bullying nor was its interaction with ethnic prejudice. In contrast, the interaction term between moral disengagement and closeness with the teacher had a significant effect on ethnic bullying. A simple slope analysis (Figure 3) evidenced that moral disengagement was positively associated with ethnic bullying perpetration for children with a low level of closeness with the teacher (*B*=0.11; *p*<0.001), whereas this association was not significant for children with a high level of closeness with the teacher (*B*=−0.01; *p*>0.05).

**Table 2:** Unstandardized regression coefficients of moral disengagement and ethnic prejudice on ethnic bullying, and the moderating effect of closeness with the teacher.

Predictors (IV)	Model 1 (DV: MD)	Model 2 (DV: EB)
Ethnic Prejudice	0.15^***^	0.04^**^
Moral Disengagement		0.05^**^
Closeness		−0.02
Ethnic Prejudice X Closeness		0.03
Moral Disengagement X Closeness		−0.08^**^
Gender	−0.02	−0.00
Grade	0.04	0.00
Immigrant Status	−0.00	0.04^**^
*R^2^*	0.05^***^	0.08^***^
*F*	7.55	5.55

IV, independent variable; DV, dependent variable; MD, moral disengagement; and EB, ethnic bullying. Gender is coded as: boys=−1 and girls=+1. Immigrant status is coded as: Italian=− 1 and immigrant=1 **p<0.01; ***p<0.001.

Results further indicated that the indirect effect of ethnic prejudice on ethnic bullying perpetration via moral disengagement was moderated by the quality of student-teacher relationship (*B*=−0.01; *SE*=0.01; 95% *CI*=−0.0274, −0.0008). Specifically, for children with a low level of closeness with the teacher, ethnic prejudice had a positive effect on ethnic bullying via moral disengagement (*B*=0.02; *SE*=0.01; 95% *CI*=0.0017, 0.0376), whereas this indirect effect was non-significant in case of high closeness (*B*=0.00; *SE*=0.00; 95% CI=−0.0094, 0.0064).^3^

## Discussion

In the present work, a moderated mediation model was proposed examining whether moral disengagement mediated the linkage between negative feelings and attitudes toward pupils from other countries and ethnic bullying perpetration, and whether closeness with the teacher impacted the direct and indirect associations between ethnic prejudice, moral disengagement, and ethnic bullying.

Results partially confirmed our hypotheses. Ethnic prejudice, in terms of its affective component, was positively related to ethnic bullying, and moral disengagement mediated this link. In addition, findings highlighted that closeness with the teacher moderated the association between moral disengagement and ethnic bullying, whereas it did not moderate the relation between ethnic prejudice and ethnic bullying. Finally, this study pointed out that the indirect effect of ethnic prejudice on ethnic bullying was stronger for pupils with low levels of closeness with their teacher. In the following paragraphs, these outcomes are discussed thoroughly.

## Direct and Indirect Effects on Ethnic Bullying

The hypothesis that ethnic prejudice was directly and positively associated with both ethnic bullying perpetration and moral disengagement was supported (H1). Consistently with previous studies that considered adolescents samples (Papotti and Caravita, 2020), the current work highlighted that the affective component of ethnic prejudice, that is experiencing aversive feelings (e.g., annoyance) toward pupils from another country, might trigger engagement in bullying also among children. In compliance with the social categorization framework (Tajfel and Wilkes, 1963; Tajfel et al., 1971), it could be speculated that children might tend to classify their schoolmates on the base of their salient cultural (e.g., spoken language) or physical (e.g., skin color) traits. This process might easily lead to “Us” vs. “Them” construal, as well as to in-group favoritism and outgroup derogation (Tajfel et al., 1971). As research showed (Kawakami et al., 2017), the use of social categories might affect intergroup relations, resulting in negative responses to the outgroups. It could be the case of this form of selective bullying that targets individual on ground of their ethnical and cultural background.

As expected, more prejudiced children might also be more morally disengaged, meaning that individuals might not feel any sense of guilt or blame when they hold negative attitudes toward ethnic outgroups, who are seen as a threat to one’s in-group. Thus, children who have hostile feelings toward outgroups might also suspend their moral principles and values that would prevent them from derogating members of the outgroup. In turn, moral disengagement was connected to bullying based on ethnicity (H2). This result is in line with works showing moral disengagement as conductive of traditional bullying behaviors (Hymel and Bonanno, 2014; Killer et al., 2019) and with those highlighting the pervasive role of moral cognitive processes in prompting ethnic bullying among children and adolescents (Caravita et al., 2019; Bayram Özdemir et al., 2020). Additionally, these findings confirmed that moral disengagement might be influenced by situational dimensions, such as the ethnicity of the target. Indeed, one of the main novelties of this study is that it assessed children’ tendency to justify aggression toward specific peers characterized by different nationalities and cultural backgrounds (Caravita et al., 2019).

Evidence for H3 about the mediation of moral disengagement between ethnic prejudice and ethnic bullying was also found. Children might be able to put their aversive feelings into action also by adopting moral strategies that would help them justify their reprehensible conduct, such as affirming that harassing someone on ground of his/her ethnic origins is not severe, but legitimate and deserved. According to the integrative social-cognitive developmental perspective on prejudice (Rutland et al., 2010), in their pathway toward bullying others because of their cultural origins, children might ground their choice both on group-based criteria, such as preserving own in-group, and on moral reasoning, such as evaluating admissible to act unfavorably toward members of the outgroups.

## The Moderating Effect of Closeness with the Teacher

In line with our expectations, closeness with the teacher moderated the relation between moral disengagement and ethnic bullying, as well as the indirect effect of ethnic prejudice on ethnic bullying via moral disengagement (H4). Although morally disengaged children tend to bully their peers independently of the relationship with teachers, we can surmise that a not close relationship may facilitate such association, whereas a harmonious one is likely to make moral disengagement ineffective in pushing pupils to bully outgroup members. It seems that moral disengagement does not necessarily lead to bullying because, when protective factors intervene, the antisocial final behavior may be avoided. In addition, a little close bond with the teacher might foster prejudiced children to turn their aversive feeling into action by facilitating their mechanisms of moral disengagement toward culturally different peers. Altogether, it is likely that, regardless of their prejudice and moral cognition, children take their teachers as an example to follow or imitate, or as a source of information to guide behavior (Bouchard and Smith, 2017), in line with the theories previously advanced and in the following discussed.

Bandura’s (1991) social-cognitive theory of morality proposes that moral agency is learned also through the environment in which individuals are embedded. As suggested by Rose et al. (2016) with preschool children, teachers are often unaware of bullying behavior, and, if they respond, they use verbal reprimands. It is possible that teachers are more ready to react to bullying behaviors enacted by those children they feel closer and to employ effective strategies with them, such as encouraging empathy for the victims or condemning the aggressive behavior, and less likely to blame the bully, which has been found ineffective in predicting bullies’ intention to stop their behavior (Garandeau et al., 2016). Teachers may also be more prone to help these children reframe the meaning of their immoral behaviors (e.g., by showing them the consequences of bad actions), or it could be that they better know how to deal with them.

Following an attachment point of view, a little close relationship with the teacher may reflect a sort of independence or avoidance on behalf of specific children, who may feel freer to behave in an undesired and antisocial way. It is possible that teachers holding a positive relationship with their students communicate them that they care and have expectations about their behavior, which may contribute to refrain from bullying (Yoon and Bauman, 2014). When bullies have a close relationship with their teachers, they may wish to comply with the rules and norms set by them, in order to appear nice at their eyes, not disappoint them, and maintain or improve such a lovely bond. Their attempt to avoid any damage to this relationship may also be associated with their worry of undermining their self-esteem, which is related to positive student-teacher relationships (van Aalst et al., 2021).

Contrary to our hypotheses, closeness with the teacher did not moderate the relation between ethnic prejudice and ethnic bullying perpetration. It is likely that children might have interiorized aversive prejudices, which may have deep roots within them and be hardly hindered by teachers (Nesdale, 2010; Geerlings et al., 2017). Also, it could be posited that rather than teachers’ relational and emotional support, it is teachers’ explicit and implicit views on cultural diversity to weaken the impact of children’s ethnic prejudice on their proneness to bully outgroup members.

Finally, although the impact of grade, gender, and immigrant status on bullying was not among our aims, we just mention that neither grade nor gender were associated with bullying in the regressions. As for grade, it can be due to the similar age of the participants, whereas the fact of not distinguishing between direct and indirect forms of bullying may account for similarities between girls and boys. Children with a migratory background were more engaged in bullying episodes, compared to their majority peers, which is in line with some findings among adolescents (Fandrem et al., 2009; Larochette et al., 2010). The need for peer acceptance and affiliation, disadvantages associated with immigrant children’s environment, and social stigma that non-natives face daily in host countries might be regarded as underlying motives for immigrant pupils to initiate aggressive behaviors (Xu et al., 2020). Alternatively, it could be just a matter of probabilities, given that, in Italian classrooms, the possible victims with different origins from one’s own are more numerous for immigrant children than for Italian children.

## Limitations, Strength Points, and Implications for Practice

This study should be interpreted in light of several shortcomings. Its cross-sectional design hindered the possibility to establish the direction of the associations among the variables. Thus, future research may adopt a longitudinal approach which would be helpful to ascertain developmental or causal pathways. Then, student-teacher relationship was detected by using only teachers’ perspective; including children’s point of view may provide a clearer picture of the interactions between pupils and their teachers. Future works are encouraged to also take into account other relevant variables associated with ethnic bullying, such as those referring to intergroup relations (e.g., group identity, intergroup contact, and ethnocultural empathy) or to other aspects of prejudice beyond the affective one (e.g., cognitive and behavioral). Similarly, they could take advantage from employing less direct assessments, such as peer reports and observations.

Some strength points can be highlighted as well, such as the employment of a comprehensive model showing the interplay of individual (ethnic prejudice and moral disengagement) and contextual factors (student-teacher interactions) that might set the stage for ethnic bullying. In addition, a large sample size was recruited, and self-reports and teacher-reports were used, which reduce shared variance. Finally, we adopted a measure of moral disengagement that specifically addressed a target with a migratory background.

These outcomes, although correlational, might indicate some practical suggestions for teachers, educators, and practitioners and would be helpful in the implementation of anti-bullying programs in multicultural primary schools. As a possible underlying mechanism of ethnic bullying is experiencing negative feelings toward culturally different children, schools are encouraged to facilitate positive intergroup contact and to involve pupils in activities that improve their perception of and their empathy toward outgroups (Sklad and Park, 2017). Also, interventions are recommended to reduce moral disengagement, helping children reconstruct their beliefs about violence, be aware of negative consequences of their acts, and enhance their sense of personal responsibility for their conduct (Hymel and Bonanno, 2014).

Programs targeting teachers are relevant to underline their central role in children’s wellbeing and to empower them. Albeit necessary, strengthening teachers’ ability to intervene in bullying situations or changing their attitudes toward bullying may not be enough; it seems paramount that teachers develop socio-emotional skills and become “mindful of their relationships with students” (Bouchard and Smith, 2017, p. 117). Trainings for teachers, specifically aimed at reinforcing this type of competence and at promoting awareness, are therefore recommended, in particular if such education is missing or not systematic. Teachers who are equipped with good socio-emotional competencies can recognize their own and their pupils’ emotions, are sensitive to their students’ needs and desires, and can respond properly, especially to those children more easily involved in bullying behaviors. Indeed, a close teacher-child relationship may foster the development of a secure and supportive context in which violence and aggression are discouraged, and students’ cooperative and relational skills promoted (Jennings and Greenberg, 2009; Bouchard and Smith, 2017).

## Data Availability Statement

The dataset presented in this article is not readily available to guarantee participants’ privacy. Participants were ensured that data would have been disseminated only in an aggregated form, that is, at a group level. Requests to access the dataset should be directed to the authors.

## Ethics Statement

The study involving human participants was reviewed and approved by the Institutional Review Board, Department of Languages and Literatures, Communication, Education, and Society, University of Udine, Italy (protocol N. CGPER-2019-12-09-05). Written informed consent to participate in this study was provided by teachers and by children’s parents or legal guardians.

## Author Contributions

MMI was involved in designing the work, collecting and analyzing the data, interpreting the results, and writing. MC supervised the project, organized the data collection, and contributed in analyzing data and writing and revising the paper. CG supervised data collection and was involved in the design and interpretation of this work as well as in revising it. NP was involved in revising the manuscript. All authors contributed to the article and approved the submitted version.

## Conflict of Interest

The authors declare that the research was conducted in the absence of any commercial or financial relationships that could be construed as a potential conflict of interest.

## Publisher’s Note

All claims expressed in this article are solely those of the authors and do not necessarily represent those of their affiliated organizations, or those of the publisher, the editors and the reviewers. Any product that may be evaluated in this article, or claim that may be made by its manufacturer, is not guaranteed or endorsed by the publisher.

## Footnotes

^1^For parsimony, throughout the paper, we often used only the term “ethnic prejudice” to refer to the affective component of ethnic prejudice, which was actually what we tested.

^2^According to the Italian law, a person is considered a foreigner if both his/her parents have a non-Italian citizenship, even if he/she is born in Italy. Consequently, a child born from an Italian parent and a foreigner parent is considered Italian, similarly to a child born from two Italian parents.

^3^Adopting an exploratory approach, we also observed whether the moderated mediation associations held for Italian and immigrant students in the same way or differently. Therefore, we added the immigrant status as another moderator of the mediation and employed the Model 17 in Process, which allows two moderators in the relations between independent variable and outcome and between mediator and outcome. Results indicated that the immigrant status neither moderated the mediation (*B* = 0.01; *SE* = 0.01; 95% *CI* = -0.0137, 0.0325) nor resulted in significant interactions with moral disengagement and prejudice (*B* = 0.02, *p* > 0.05, and *B* = 0.02, *p* > 0.05, respectively), highlighting that the processes involved worked in the same way for all children, being them Italians or immigrants.
